# Inhibition of NHE1 transport activity and gene transcription in DRG neurons in oxaliplatin-induced painful peripheral neurotoxicity

**DOI:** 10.1038/s41598-023-31095-9

**Published:** 2023-03-09

**Authors:** Marianna Dionisi, Beatrice Riva, Marta Delconti, Cristina Meregalli, Alessia Chiorazzi, Annalisa Canta, Paola Alberti, Valentina Carozzi, Eleonora Pozzi, Dmtry Lim, Armando A. Genazzani, Carla Distasi, Guido Cavaletti

**Affiliations:** 1 Department of Pharmaceutical Sciences, Università del Piemonte Orientale, Via Bovio 6, 28100 Novara, Italy; 2 Experimental Neurology Unit, School of Medicine and Surgery, University of Milano-Bicocca, Monza, Italy

**Keywords:** Peripheral nervous system, Transporters in the nervous system, Cell signalling, Mechanisms of disease

## Abstract

Oxaliplatin (OHP)-induced peripheral neurotoxicity (OIPN), one of the major dose-limiting side effects of colorectal cancer treatment, is characterized by both acute and chronic syndromes. Acute exposure to low dose OHP on dorsal root ganglion (DRG) neurons is able to induce an increase in intracellular calcium and proton concentration, thus influencing ion channels activity and neuronal excitability. The Na^+^/H^+^ exchanger isoform-1 (NHE1) is a plasma membrane protein that plays a pivotal role in intracellular pH (pH_i_) homeostasis in many cell types, including nociceptors. Here we show that OHP has early effects on NHE1 activity in cultured mouse DRG neurons: the mean rate of pH_i_ recovery was strongly reduced compared to vehicle-treated controls, reaching levels similar to those obtained in the presence of cariporide (Car), a specific NHE1 antagonist. The effect of OHP on NHE1 activity was sensitive to FK506, a specific calcineurin (CaN) inhibitor. Lastly, molecular analyses revealed transcriptional downregulation of NHE1 both in vitro, in mouse primary DRG neurons, and in vivo, in an OIPN rat model. Altogether, these data suggest that OHP-induced intracellular acidification of DRG neurons largely depends on CaN-mediated NHE1 inhibition, revealing new mechanisms that OHP could exert to alter neuronal excitability, and providing novel druggable targets.

## Introduction

Oxaliplatin (OHP), a third-generation platinum compound, is the cornerstone drug for colorectal cancer treatment, one of the commonest solid neoplasms. Notably, the majority of patients receiving OHP develop peripheral neurotoxicity (OIPN, oxaliplatin-induced peripheral neurotoxicity), a severe and challenging complication due to primary dorsal root ganglia (DRG) neurons damage^1^, and characterized by both an acute and a chronic syndrome; the former is a transient hyperexcitability syndrome characterized by cold-induced paresthesias and cramps lasting 48–72 h after OHP administration, whereas the latter is a dose-dependent chronic sensory neuronopathy with impaired distal sensory perception and ataxia^2^.

Extensive studies have shown that the pathological mechanisms of OIPN are complex, multi-factorial and involve several cellular and molecular processes, such as changes in ion channel regulation, mitochondrial dysfunction and oxidative stress, altered calcium homeostasis, immune responses, neuroinflammation and axon neurodegeneration^3–5^. Furthermore, the OHP-induced intracellular pH acidification in DRG sensory neurons has been described as one of the early effects of the drug^6,7^. The reduction of the cytosolic [H^+^] in neurons is caused by the formation of neuronal hemoglobin-OHP adducts, which decrease cytosolic proton buffering capacity^8^. Moreover, the intracellular pH (pH_i_) change alters the electrical properties of neurons by sensitizing TRPA1 and modulating the activity of TREK channels^7,9^. On the other hand, in addition to buffering, the steady-state pH_i_ depends on the balance between the rate of H^+^ production from cell metabolism and the rates of acid extrusion and acid loading via alkalizing and acidifying membrane transport systems^10,11^. Among them, the Na^+^/H^+^ exchangers (NHEs) play a pivotal role in maintaining pH homeostasis in cell organelles, cytosol and tissues.

In mammals, thirteen genes are currently known to encode thirteen isoforms, classified as SLC9A1-9 (NHE1-9), SLC9B1-2 (NHA1-2), and SLC9C1-2. With only a few exceptions, the NHE isoform 1 (NHE1/SLCA1) is ubiquitously expressed, commonly located on the plasma membrane, and regulates, in addition to pH, several physiological functions such as cell volume, proliferation, scaffolding, survival and motility.

Multiple mechanisms regulate NHE1 activity, including changes in intracellular Ca^2+^ and H^+^ concentration and interaction with several binding proteins such as protein kinases and phosphatase^12^.

Likewise, a number of studies carried out in peripheral sensory neurons have shown a role of NHE1 in nociception^13–18^. In particular, the blockade of NHE1 activity increases nociceptive behavior in acute pain models^15,18^ and promotes mechanical allodynia and hyperalgesia in formalin-induced nociception^14^. Nevertheless, the role of the NHE1 in neuropathic pain and in particular in OIPN is poorly characterized and is still controversial.

Therefore, in the present study we investigated the role of NHE1 in OHP-induced cytosolic acidification. We found that OHP, but not 5-fluorouracil (5-FU), a non-neurotoxic anticancer agent often administered with OHP in patients^19^, inhibits the activity of the NHEs in primary mouse DRG neurons. Moreover, we provide a pharmacological evidence that suggests a role of the Ca^2+^/calmodulin-dependent phosphatase calcineurin (CaN) in the OHP-induced regulation of the NHE1 activity. Finally, we reported that OHP downregulates NHE1 transcription both in vitro in mouse DRG neurons and in vivo in an OIPN rat model.

## Results

### Effect of OHP on intrinsic intracellular buffering power

**Figure 1:**
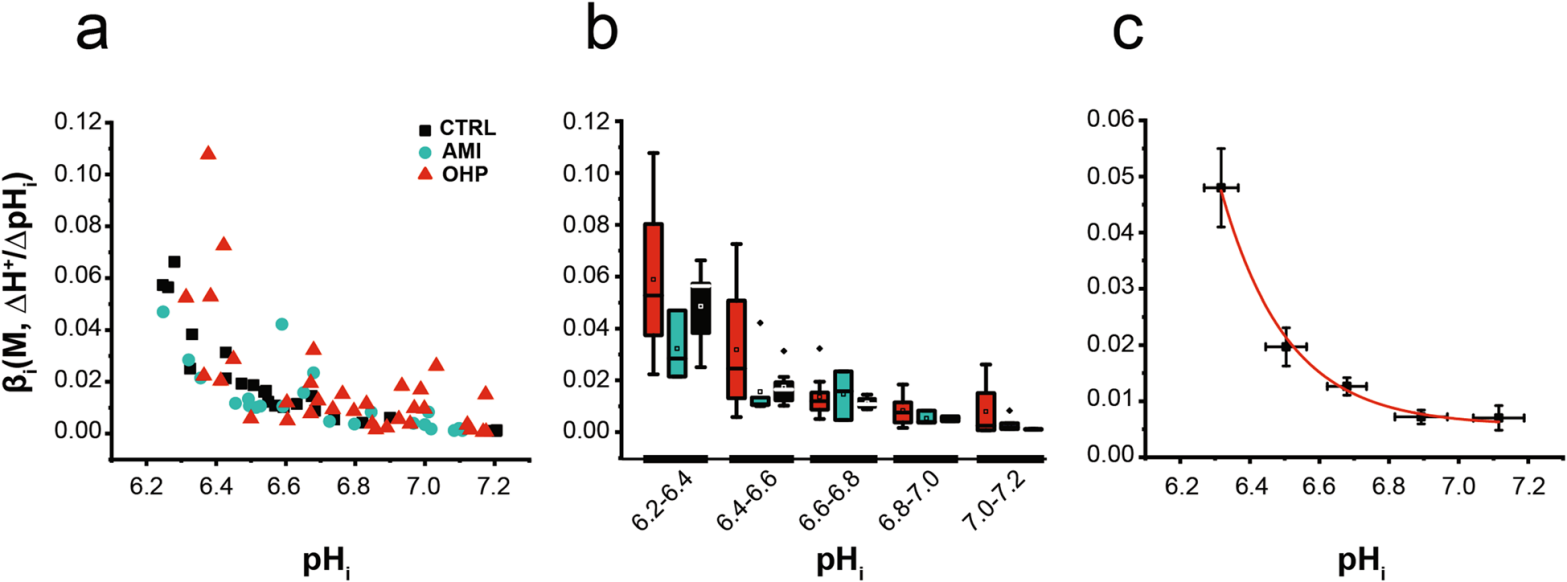
Intrinsic buffering power in DRG neurons. (**a**) Intrinsic buffering power (β_i_) as a function of pH_i_. β_i_ values were estimated, in single untreated DRG neurons or treated with OHP and AMI, from Δ pH_i_ measured upon removal of NH_4_Cl (mM) pulses in Na^+^-free solution. Data were collected from 3 to 6 different cultures for each condition. (**b**) Mean (square), median (line across the box), interquartile range (box), maximum and minimum (Hyphens over and under the box) of β_i_ values from (**a**) at five ranges of pH_i_ for untreated (CTRL), AMI- and OHP-treated neurons. (**c**) Mean and SEM of pooled β_i_ values from the different experimental conditions at the five ranges of pH_i_. Solid red line represents a single exponential decay fitting curve, namely: β_i_ = 0.006 + 0.051*exp( − (pH_i_ − 6.28)/0.185).

As previously reported, OHP is able to influence intracellular pH homeostasis of DRG neurons by inducing a long-lasting decrease in pH_i_ as early as 30 min^6,7^ and by forming adducts with neuronal hemoglobin, a key protein buffer^8^. Nevertheless, a characterization of the effect that OHP exerts on the intrinsic buffering power (β_i,_), the intracellular buffer component mainly supported by the imidazole group of histidine residues and phosphate, is still lacking. For this reason, we performed a first series of experiments to investigate whether OHP affects β_i_. Cells were acid loaded by exposure to 10, 20 or 40 mM NH_4_Cl, followed by its sudden withdrawal and β_i_ was estimated by the subsequent decrease of pH_i_. Figure 1a shows the calculated βi values plotted versus pH_i_ from untreated neurons (CTRL) or neurons incubated with OHP (0.1 μg/ml) for 0.5–1.5 h. Furthermore, to assess whether a pH_i_ compensation occurs during the acid load affecting the measurement accuracy, we calculated βi values from a set of cells acid loaded in the presence of 10 μM amiloride (AMI, Fig. 1a), a blocker of NHE^20^. Figure 1b compares the mean and the median β_i_ values at five ranges of pH_i_.

Finally, considering the lack of statistical significance between the experimental conditions investigated for each pH range considered (Kruskal–Wallis ANOVA), we have pooled and averaged all the β_i_ values for each range. In this way, as shown in Fig. 1c, the relationship between the β_i_ and pH_i_ is well fitted by an exponential decay.

### Effect of OHP, 5-FU and FK506 on NHE1 activity

**Figure 2:**
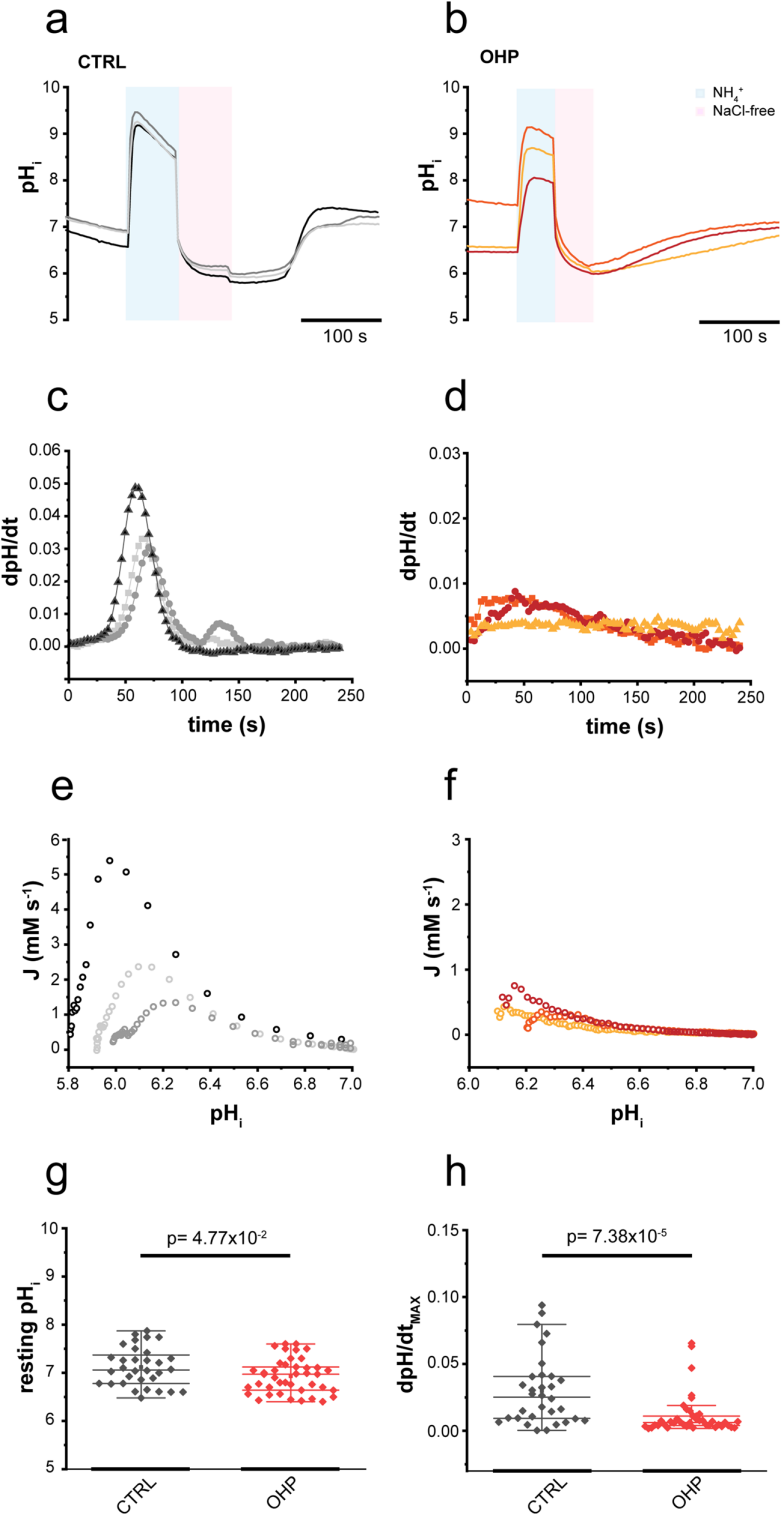
Effect of OHP on resting pH_i_ and NHE1 activity. pH_i_ recovery from an acid load in three untreated DRG neurons (**a**) and in neurons incubated for 1 h with 0.1 μg/ml of OHP (**b**). Panels (**c**) and (**d**) represent the instantaneous pH_i_ rate of change versus time of traces in (**a**) and (**b**), which were used to compute the pH_i_ dependence of the total acid extrusion J (**e**, **f**). Statistical plots (Mann–Whitney U test) compare control (black) and OHP-treated (red) resting pH_i_ (**g**) and the maximum rate of recovery dpH/dt_MAX_ (**h**).

To evaluate the impact of OHP on acid–base transporters activity, we evaluated the pH_i_ recovery from acute intracellular acid load in cells exposed to a nominally CO_2_/HCO_3_^−^ free solution, a condition in which acid extrusion is primarily mediated by Na^+^/H^+^ exchangers^21^. Figure 2a shows three representative examples of the time course of the pH_i_ recovery recorded from untreated DRG neurons: cells promptly and rapidly recovered from the acid load after the extracellular Na^+^ was added back. Conversely, as shown in Fig. 2b, a short incubation time (0.5–1.5 h) with OHP markedly slowed the pH_i_ recovery and decreased the resting pH_i_ (MD_CTRL_ = 7.06, IQR_CTRL_ = [6.78; 7.39], n = 32; MD_OHP_ = 6.97, IQR_OHP_ = [6.64; 7.14], n = 41; *p*-value = 0.047, Mann–Whitney U test).

To quantify the differences in NHE activity under the two conditions, we calculated, for each trace, the instantaneous rate of change dpH_i_/dt and the maximal rate of recovery dpH/dt_MAX_ (Fig. 2c,d). We found that OHP significantly decreased the dpH/dt_MAX_ (MD_CTRL_ = 0.025, IQR_CTRL_ = [0.009; 0.041], n = 32; MD_OHP_ = 0.006, IQR_OHP_ = [0.004; 0.011], n = 41; *p*-value = 7.38 × 10^−5^, Mann–Whitney U test). Furthermore, we determined the pH_i_ dependence of the total acid extrusion J as the product of dpH_i_/dt and β_I_ and we found that OHP incubation markedly reduced the J values in the acidic pH range (Fig. 2e,f). The results are summarized in the statistical plots of Fig. 2g (resting pH_i_) and 2 h (maximum rate of recovery dpH/dt_MAX_).

**Figure 3:**
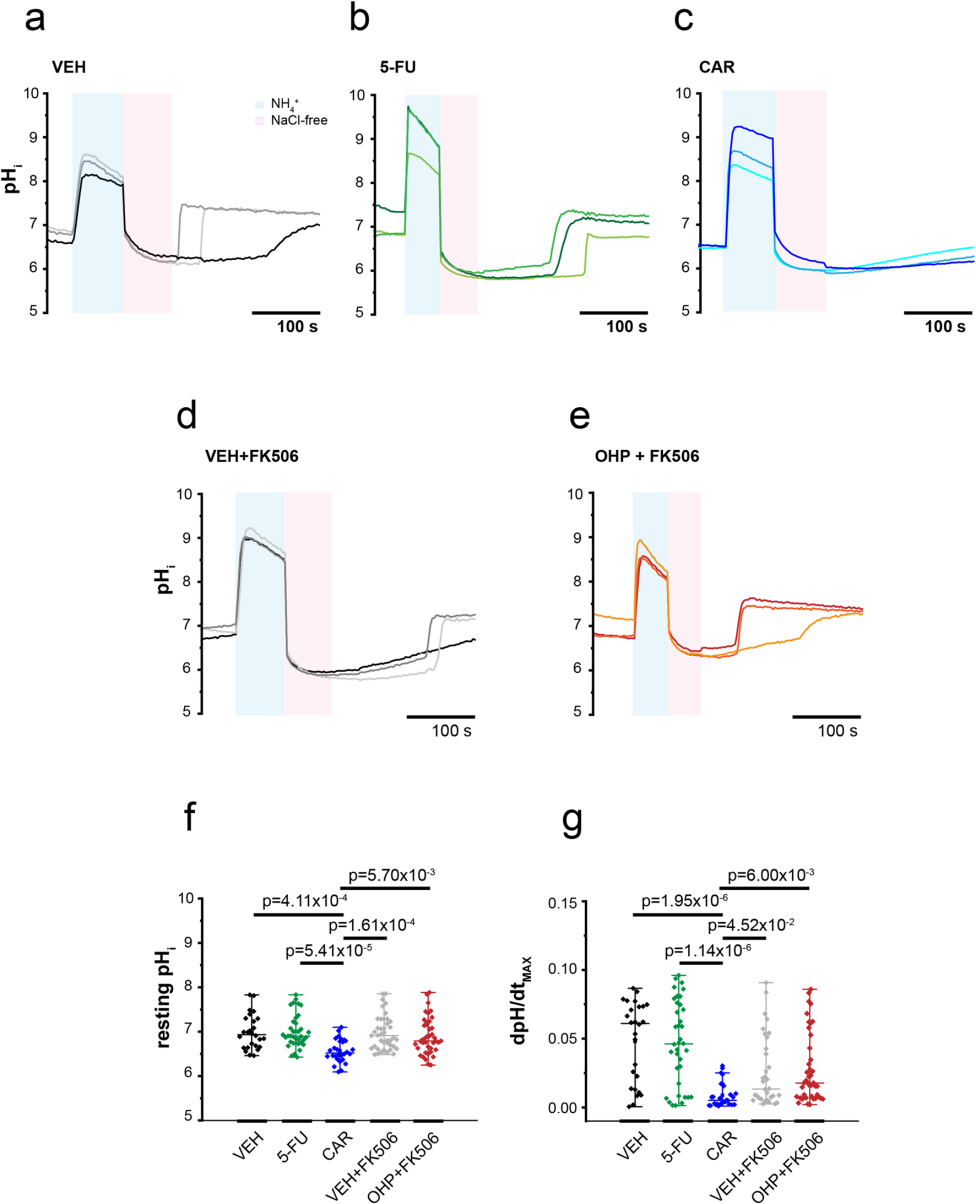
Effect of 5-FU and FK506 on steady state pH_i_ and NHE1 activity. Three exemplary traces of pHi recovery from an acid load in DRG neurons incubated with: VEH (**a**), 5-FU (**b**), CAR (**c**), FK506 (**d**), FK506 and OHP (**e**). Statistical plots (Kruskal–Wallis and Dunn’s test) comparing resting pH_i_ (**f**) and the maximum rate of recovery dpH/dt_MAX_ (**g**) values obtained in the five experimental conditions.

Next, we tested whether 5-fluorouracil (5-FU), another anticancer agent often administered with OHP in tumor patients, but devoid of any peripheral neurotoxicity in clinical use as well as in this experimental study (see below), was also able to affect NHE activity and consequently to produce a cytosolic acidification in DRG neurons. To this end, we measured the resting pH_i_ and calculated dpH/dt_MAX_ from recordings obtained in neurons incubated with vehicle alone (DMSO 0.1% v/v, Fig. 3a) or 5-FU (500 nM, Fig. 3b). We found that 5-FU does not change either the steady state pH_i_ (MD_VEH_ = 6.93, IQR_VEH_ = [6.65; 7.30], n = 26; MD_5-FU_ = 6.90, IQR_5-FU_ = [6.74; 7.22], n = 36; Kruskal–Wallis and Dunn’s test) or NHE activity (MD_VEH_ = 0.061, IQR_VEH_ = [0.013; 0.074], n = 26; MD_5-FU_ = 0.046, IQR_5-FU_ = [0.011; 0.076], n = 36; Kruskal–Wallis and Dunn’s test). Moreover, as shown in Fig. 3c, cariporide (CAR, 30 μM), a specific and powerful inhibitor of the NHE isoform 1^22,23^, significantly decreased both the resting pH_i_ (MD_CAR_ = 6.52, IQR_CAR_ = [6.40; 6.79], n = 28; *p*-value = 4.11 × 10^−4^ vs VEH Kruskal–Wallis and Dunn’s test) and the dpH/dt_MAX_. (MD_CAR_ = 0.005, IQR_CAR_ = [0.002; 0.014], n = 28; *p*-value = 1.95 × 10^−6^ vs VEH Kruskal–Wallis and Dunn’s test). Overall, our data suggests that NHE1 mediates a large component of the recovery from an acidic pH_i_ in DRG neurons and is heavily involved in the OHP-dependent pH_i_ acidification.

Finally, to examined whether the Ca^2+^ phosphatase calcineurin (CaN) was involved in the inhibition of NHE1, we performed a set of experiments by incubating cells for 0.5–1.5 h with the CaN specific inhibitor FK506^24^ (1 µM) alone (Fig. 3d) or in the presence of OHP (Fig. 3e). We found that the resting pH_i_ and dpH/dt_MAX_ values were comparable between neurons pre-incubated with vehicle (DMSO, see above) alone or with FK506 (resting pH_i_, MD_FK506_ = 6.91, IQR_FK506_ = [6.65; 7.26], n = 32; dpH/dt_MAX_, MD_FK506_ = 0.013, IQR_FK506_ = [0.006; 0.049], n = 32; Kruskal–Wallis and Dunn’s test). On the other hand, the treatment with FK506 significantly counteracted the NHE1 inhibition induced by OHP (MD_OHP+FK506_ = 0.018, IQR_OHP+FK506_ = [0.007; 0.048], n = 41; Kruskal–Wallis and Dunn’s test) and the steady state pH_i_ acidification (MD_OHP+FK506_ = 6.79, IQR_OHP+FK506_ = [6.61; 7.18], n = 41; Kruskal–Wallis and Dunn’s test). The results are summarized in the statistical plots of Fig. 3f,g. Overall, these results suggest that OHP inhibits NHE1 by mechanisms involving CaN activation.

### OHP, but not 5-FU, alters NHE1 expression in DRG in vitro and in vivo

**Figure 4:**
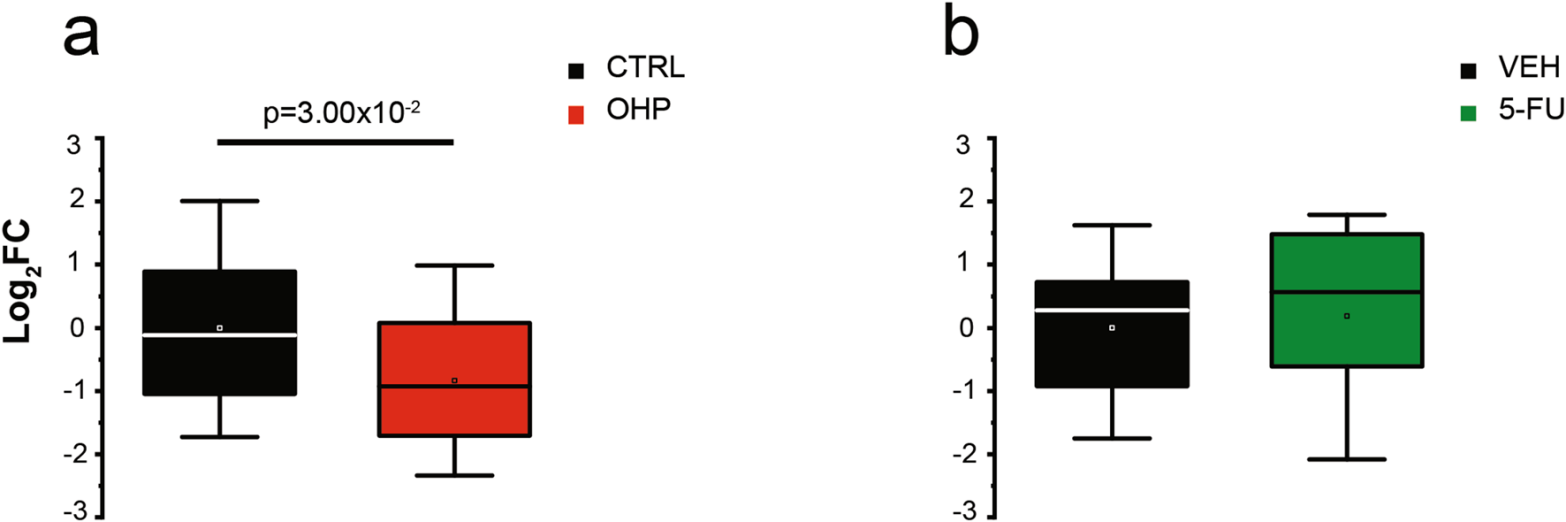
OHP affects the transcription of NHE1 in DRG cultures. Mean (square), the median (line across the box), and interquartile range (box) of treated vs. control or vehicle log_2_ FC of gene expression, as measured through real-time quantitative PCR (RT-qPCR). (**a**) OHP-induced changes in mRNA level were measured in cultured DRG neurons from mice, after 6 h of treatment. (**b**) mRNA expression from DRG neurons was also measured after 5-FU treatment.

We evaluated the effect of OHP and 5-FU on NHE1 transcripts levels in DRG cells. A first set of experiments was carried out in DRG cultures from mice and the mRNA levels of NHE1 exchanger were measured after a 6 h-treatment with the antineoplastic drugs. As shown in Fig. 4a, OHP treatment resulted in a significant decrease in NHE1 gene expression (log_2_ FC, MD_CTRL_ = − 0.12, IQR_CTRL_ = [− 1.08; 1.15]; MD_OHP_ = − 0.93, IQR_OHP_ = [− 1.71; 0.21], n = 4 biological replicates, each in technical triplicate; *p*-value = 0.030, Mann–Whitney U test). Conversely, the treatment with 5-FU did not induce any effect (Fig. 4b, log_2_ FC, MD_VEH_ = 0.28, IQR_VEH_ = [− 0.94; 0.75]; MD_5-FU_ = 0.57, IQR_5-FU_ = [− 0.72; 1.50]; n = 3 biological replicates, each in technical triplicate; Mann–Whitney U test). This evidence suggests that OHP, not only negatively modulates with high specificity NHE1 activity, but also regulates NHE1 gene transcription, in vitro.

**Figure 5:**
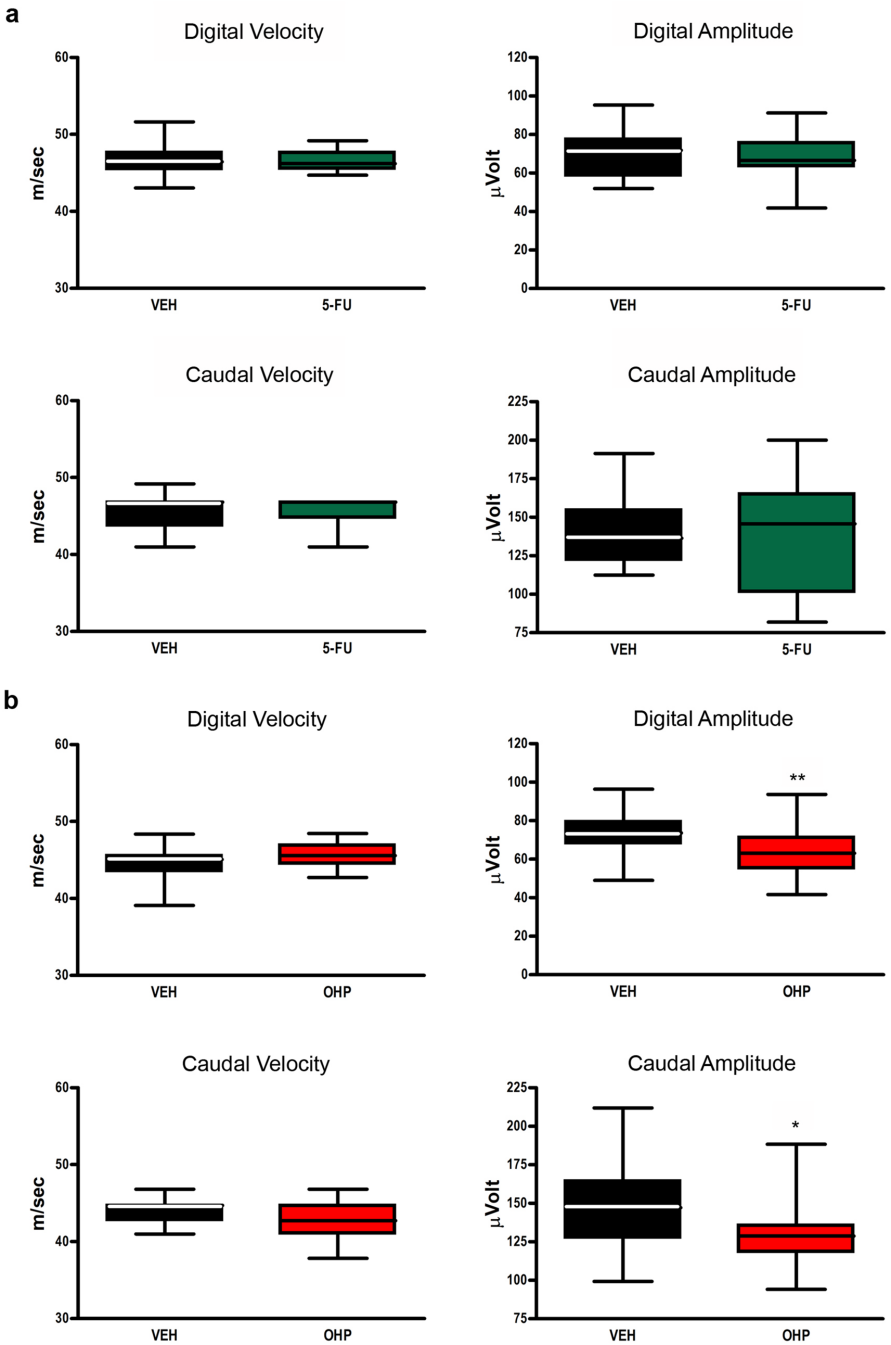
Nerve conduction studies at the end of the treatment. (**a**) neurophysiological parameters of both caudal and digital nerves in the 5-FU experiment: no statistical significance was demonstrated for any tested parameter. (**b**) neurophysiological parameters for OHP experiment; statistical significance for Mann–Whitney U test is shown (*p < 0.05; **p < 0.01).

Considering the above outcomes, we decided to investigate the effect of OHP and 5-FU on gene expression of NHE1 antiporters in an in vivo rat model for OIPN^25^. To verify neuropathy ensued, we relied on nerve conduction studies at the end of treatments, with a consolidated protocol able to reproduce the gold standard in the clinical setting. The body weight changes show that OHP induces the most relevant reduction of body weight compared to CTRL and 5-FU treated animals (see Supplementary Table S1). One OHP-treated mouse was sacrificed before the last administration, while no distress was evident in the remaining animals. In the 5-FU experiment, it was demonstrated that this drug is not neurotoxic (Fig. 5a). Instead, OHP-treated animals showed a neurophysiological pattern compatible with a mild neuronopathy with secondary axonopathy as evidenced by caudal nerve investigation (Fig. 5b), matching data from clinical settings^2^.

**Figure 6:**
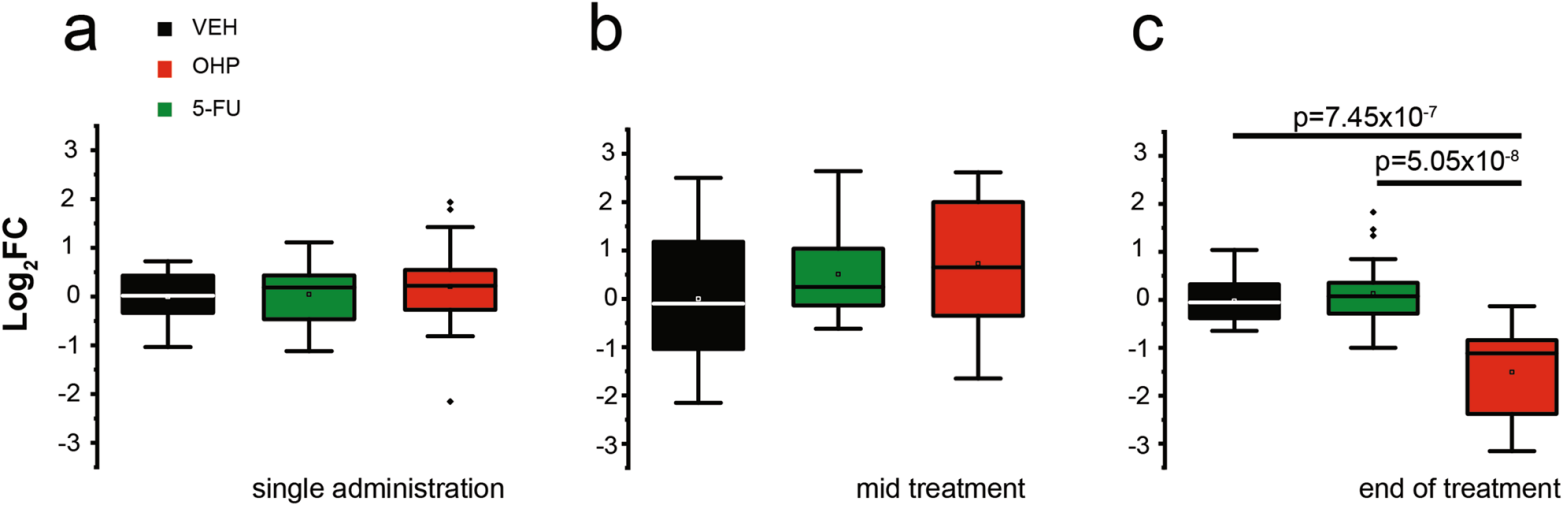
OHP affects the transcription of NHE1 in the DRG in vivo. Mean (square), the median (line across the box), and interquartile range (box) of treated vs. VEH log_2_ FC of gene expression, as measured through real-time quantitative PCR (RT-qPCR). Changes in mRNA levels were measured in DRG from rats upon in vivo acute, (single administration (**a**), log_2_ FC, MD_CTRL_ = 0.02, IQR_CTRL_ = [− 0.35; 0.44]; MD_5-FU_ = 0.19, IQR_5-FU_ = [− 0.51; 0.44]; MD_OHP_ = 0.22, IQR_OHP_ = [− 0.27; 0.55]; Kruskal–Wallis and Dunn’s test and chronic treatments with OHP- or 5-FU, compared with their VEH (mid treatment (**b**), log_2_FC, MD_CTRL_ = − 0.10, IQR_CTRL_ = [− 1.14; 1.42]; MD_5-FU_ = 0.25, IQR_5-FU_ = [− 0.14; 1.17]; MD_OHP_ = 0.65, IQR_OHP_ = [− 0.40; 2.07] n = 4 biological replicates, each in technical triplicate; end of treatment (**c**), log_2_ FC, MD_CTRL_ = − 0.05, IQR_CTRL_ = [− 0.39; 0.32]; MD_5-FU_ = 0.07, IQR_5-FU_ = [− 0.29; 0.36]; MD_OHP_ = − 1.12, IQR_OHP_ = [− 2.49; − 0.81]; *p*-value = 7.45 × 10^−7^ OHP vs VEH; *p*-value = 5.05 × 10^−8^ OHP vs 5-FU; n = 4 biological replicates, each in technical triplicate; Kruskal–Wallis and Dunn’s tests). In all panels, VEH are normalized to zero.

The NHE1 mRNA levels were measured 24 h after the last treatment with vehicle alone or drugs following a single administration (Fig. 6a), mid treatment (Fig. 6b) and end of treatment (Fig. 6c). We observed a significant decrease in the mRNA levels of NHE1 transporters in DRG neurons only after 6 weeks of treatment with OHP (Fig. 6c) (log_2_ FC, MD_CTRL_ = − 0.05, IQR_CTRL_ = [− 0.39; 0.32]; MD_5-FU_ = 0.07, IQR_5-FU_ = [− 0.29; 0.36]; MD_OHP_ = − 1.12, IQR_OHP_ = [− 2.49; − 0.81]; *p*-value = 7.45 × 10^−7^ OHP vs CTRL; *p*-value = 5.05 × 10^−8^ OHP vs 5-FU; Kruskal–Wallis and Dunn’s test).

## Discussion

Alterations in pH_i_ have profound effects on nerve excitability and have been proposed as factors or co-factors of many dysfunctions in both the peripheral and central nervous systems^21,26–28^. In mice DRG sensory neurons, we have shown that OHP induces early pH_i_ alterations in vitro and in vivo that modify the activity of thermosensitive calcium-permeable TRPA1 and TRPV1 channels and potassium K2P channels^7,9^, key regulators of sensory neurons excitability^29^. This study presents novel findings on the OHP ability to alter NHE1 activity after short incubation times, as well as, the first measurement of the intrinsic buffering power and its pH_i_ dependence in mice DRG neurons.

As for β_i_, the resulting values are in general in good agreement with data obtained from other cell types^30–34^ with a pH dependence well described by a single exponential decay^30^. Although, recently, it has been shown that therapeutic concentrations of OHP is able to form adducts with hemoglobin in DRG from mice treated with a single intravenous injection of the chemotherapy drug^8^, in cultured DRG neurons we observed that the incubation for 0.5–1.5 h with OHP does not produce significant changes on β_i_. A number of factors may account for this discrepancy, including the predominant role of intracellular buffers other than hemoglobin in sensory neurons.

To our knowledge, no data are available on the role of platinum-based drugs on NHE1 activity in neuronal cells. However, it has been reported that cisplatin acutely affects the intracellular pH in human colon carcinoma and in HeLa cell lines by inhibiting NHE1 activity through a mechanism independent of adducts formation^35–37^.

Notably, NHE1 hyperactivation is observed in a number of cancer types where it reverses the transmembrane pH gradient lowering pH_e_, a key step in oncogenic transformation and a permissive signal for cell proliferation^38,39^. Thus, while OHP inhibition of NHE1 may contributes to the therapeutic action by re-establishing the pH gradient towards a physiological condition unfavorable to neoplastic cells, in sensory neurons it will produce an early intracellular acidification leading to the activation of a cascade of adverse effects, ranging from the altered excitability and synaptic transmission to axon degeneration and cell death^11,27,40–42^.

Interestingly, we found that OHP downregulates NHE1 transcription both in vitro in mouse DRG neurons after incubation for 6 h and in vivo in a rat model of OIPN after 6-week treatment, in which drug administration was proved to be neurotoxic. Overall, these results may indicate the potential role of NHE1 in both the acute transient phase and the chronic phase of OIPN. Indeed, our results confirm the evidence obtained previously by Castaneda-Corral et al.^14^ in a rat experimental model of chronic pain, where formalin injection decreased NHE1 protein expression in the dorsal spinal cord and DRG in a time-dependent manner. An important unresolved question is whether a correlation exists between the acute dysregulation of intracellular pH by OHP-dependent NHE1 inhibition and the downregulation of mRNA levels observed at the end of the treatment. In this regard, it should be noted that NHE1 is also involved in the direct regulation of a plethora of cellular processes through scaffolding interactions mediated by its cytosolic tail in a way independent of its ion transporter function^12^. Further studies are therefore needed to clarify this point.

Finally, this study suggests an involvement of calcineurin in the OHP-dependent regulation of NHE1 activity. Among the physiological signals and mechanisms regulating NHE1 activity, multiple phosphorylation/dephosphorilation events at the cytoplasmic C-terminal domain are associated with transporter activation/inhibition^12^. CaN directly binds to the cytoplasmic domain of NHE1 and recent findings have shown that it specifically dephosphorylates NHE1 phosphothreonine 779, reducing NHE1-mediated acid extrusion^43^. CaN is activated by increased intracellular Ca^2+^-concentrations and, in DRG neurons, it has been shown that acute application of OHP triggers a Ca^2+^-release that is dependent on type 1 histamine receptors^6^. Furthermore, an alternative CaN activation pathway could involve the operation in the reverse mode of the Na^+^/Ca^2+^ exchanger isoform 2, resulting in cytosolic calcium accumulation in OHP treated neurons^44^. Lastly, in a recent paper the OHP-induced neuropathic pain was correlated to the activation of the CaN/NFAT pathway in a rat model^45^. As for CaN, the authors showed that FK506 injection in CIPN rats raised after 1 h the value of paw withdrawal threshold that had decreased after 14-day of OHP treatment.

In conclusion, our findings provide new insights of the pH_i_ regulation involving NHE1 that can be exploited to enhance strategies for the prevention and treatment of OIPN.

## Materials and methods

### Animals

BALB/c male mice aged 5–10 weeks and Wistar male rats weighing 250–275 g upon arrival (Envigo, Bresso, Italy) were employed for DRG culture preparation and in vivo studies, respectively. Animals were maintained as previously reported^3^. Care and husbandry of animals were in conformity with the institutional guidelines in compliance with national (D.L. n. 26/2014) and international laws and policies (EEC Council Directive 86/609, OJ L358, 1, Dec.12, 1987; Guide for the Care and Use of Laboratory Animals, U.S. National Research Council, 1996). The study plan and the procedures were approved by the University of Piemonte Orientale and University of Milano-Bicocca (authorization number 0041156/21) and authorized by the Italian Ministry of Health (authorization number DB064.N.TGU and 449/2020-PR). All mice were euthanized under deep isoflurane-induced anesthesia for cell cultures and rats with CO_2_ for in vivo experiments. All methods were carried out in accordance with relevant guidelines and regulations and the authors complied with the ARRIVE guidelines^46^.

### Chemicals

For in vitro studies, OHP (Sigma–Aldrich Inc., Milano, Italy), cariporide (Sigma–Aldrich Inc., Italy), amiloride (Sigma–Aldrich Inc., Italy), 5-FU (Sigma–Aldrich Inc., Italy), FK506 (Bio-Techne, Milano, Italy), nigericin (Life Technologies, Monza, Italy) and valinomycin (Life Technologies, Monza, Italy) were used. These compounds, with the exception of OHP (reconstituted in 100% water) and FK506 (reconstituted in 100% ethanol), were dissolved in 100% dimethyl sulfoxide (DMSO) and stored at − 20 °C, according to the manufacturers’ specifications. For in vivo studies, OHP (Accord, Healthcare Italia) and 5-FU (Accord, Healthcare Italia) compounds were used from stock solutions to achieve the final concentrations. For each experiment, working concentrations of these drugs were freshly prepared by diluting them in their relative vehicle.

### Isolation, culture, and treatment of mouse DRG neurons

DRG from adult BALB/c mice were excised and cultured as previously described^7^. Briefly, DRG from cervical to sacral (up to S2) level were bilaterally excised, collected and accurately de-sheathing in a dish containing cold F12 (Nutrient Mixture F12 Ham) medium (Sigma–Aldrich Inc., Italy). After incubation at 37 °C (1 h) with collagenase from Clostridium hystoliticum 0.125% (Sigma–Aldrich Inc., Italy), DRG were dissociated. Cells were plated on laminin (Sigma–Aldrich Inc., Italy) coated glass coverslips (24 mm) and cultured at 37 °C with 5% CO_2_ for 48 h in Bottenstein and Sato medium (BS)^7^ supplemented with Recombinant Human β-NGF, Recombinant Murine GDNF and Recombinant Human NT3 (Peprotech, Rocky Hill, NJ, USA). The administration of OHP (0.1 μg/ml) and 5-FU (500 nM) was performed 48 h after the isolation of DRG neurons and all the experiments were performed from 48 to 54 h of culture.

### In vivo studies

Sixty-three Wistar male rats were randomized into three treatment groups, 7 animals each for three time points (single administration, mid treatment and end of treatment). In particular, rats were treated either with OHP (5 mg/kg i.v.) or 5-FU (50 mg/kg i.v.) once to reproduce features of the acute OIPN syndrome and repeatedly (once a week for two or four times with 5-FU or twice for three or six weeks with OHP, same doses) to establish the chronic OIPN condition. A vehicle group was treated with glucose solution 5% (VEH). 24 h after every time points, the animals were sacrificed for sample collection, specifically ganglia from cervical to sacral (up to S2) level were bilaterally excised under a dissection microscope. Mortality was evaluated daily and body weight changes were measured twice a week during the treatment period, as reported in Supplementary Table S1.

Nerve conduction studies were performed as recently described^25,47^ to assess the digital and the caudal nerve sensory action potential (SAP). Recordings were performed on all the animals at the end of treatment to assess the onset of neuropathy. Electromyography apparatus Matrix Light (Micromed, Mogliano Veneto, Italy) and stainless-steel needle electrodes (Subdermal EEG needle, Ambu™, Ballerup, Denmark) were used. All procedures were performed under standard conditions under deep isoflurane anesthesia, and animal body temperature was monitored and kept constant at 37 ± 0.5 °C with a thermal pad, electronically connected to a thermal rectal probe (Harvard Apparatus, Holliston, US). The optimal setting of stimulation for each nerve was reached following the subsequent protocol with an orthodromic stimulation. *Caudal nerve* SAP: reference and active recording electrodes at 1 and 2 cm from the base of the tail, respectively; cathode and anode at 5 and 6 cm from the base of the tail, respectively; ground electrode at midway between the cathode and the active recording electrodes. *Digital nerve* SAP (left hind-paw): reference and active recording electrodes in front of the patellar bone and close to ankle bone, respectively; cathode and anode at the base of the fourth toe and at the tip of it, respectively; ground electrode in the sole. The peak-to-peak amplitude was considered. For the sensory nerve conduction velocity (SCV) calculation, onset of SAPs was considered. Filters were kept between 20 Hz and 3 kHz, sweep was kept at 0.5 ms.

### Measurement of intracellular pH

We measured intracellular pH in single cells by live-cell ratiometric imaging experiments using the fluorescent pH- sensitive BCECF dye^48^. DRG cultures were incubated with 1 µM BCECF-AM (Life Technologies, Italy) for 20 min at room temperature in Tyrode standard solution (TS) of the following composition in mM: NaCl 154; KCl 4; CaCl_2_ 2; MgCl_2_ 1; 4-(2-hydroxyethyl)-1-piperazine ethane sulfonic acid (HEPES) 5; glucose 5.5; NaOH to pH 7.4. After washing in TS, cells were placed under a Leica DMI6000 epifluorescent microscope equipped with S Fluor × 40/1.3 objective and BCECF was alternatively excited at 490 nm and 450 nm (monochromator Polychrome IV, Till Photonics, Germany) while recording at emission wavelengths > 525 nm. Data was acquired every 3 s (Hamamatsu, Japan) with MetaFluor software (Molecular Devices, Sunny-vale, CA, USA). Image time series were analysed with ImageJ (Rasband W.S., NIH, Bethesda MD) and OriginPro 9.1 (OriginLab, USA) softwares to obtain the background subtracted ratio of the mean pixel intensity within a region of interest encompassing a neuronal cell body. The ratios were converted into the pH_i_ value by the high K^+^/nigericin technique. At the end of the experiment we obtained for each neuron three calibration points at pH values of 5.5, 6.5 and 7.5 (Calibration Buffer Kit, Life Technologies) that in almost all cells were best fitted by a straight line, whose parameters were used to convert ratios to pH values.

### Measurement of intrinsic buffering power β_i_ and pH_i_ recovery from acid load

The intrinsic buffering power was measured using the NH_4_Cl prepulse technique^49,50^ in cells superfused with TS. First, cells were exposed for 75–100 s to a solution containing (in mM): NH_4_Cl 40 (20 or 10), N-methyl-d-glucamine 100 (120 or 130) HEPES 20, glucose 5.5, KCl 4, CaCl_2_ 2, MgCl_2_ 1, HCl to pH 7.4 to induce an intracellular alkalinization. Next, the solution was substituted with a 0 Na^+^ solution of the following composition: *N*-methyl-d-glucamine 140, HEPES 20, glucose 5.5, KCl, CaCl_2_ 2, MgCl_2_ 1, HCl to pH 7.4 to cause a stable intracellular acidification. The intrinsic buffering power β_i_ was calculated by dividing the amount of [NH_4_^+^]_i_ added to the cell by the decrease in pH_i_ (for full details see^49,50^). The time course of pH_i_ recovery was recorded following the replacement of 0 Na^+^ solution with TS^48,49^. Traces were slightly smoothed (adjacent-averaging method) to remove noise and the instantaneous rate of change was computed from the pH_i_(t) first order derivative. The Na^+^/H^+^ exchanger activity was then quantified as the maximum rate of change dpH/dt_MAX_. Data analysis and curve fitting (Levenberg–Marquardt algorithm) were done using OriginPro 9.1 (OriginLab, USA).

### Real-time quantitative PCR (RT-qPCR)

For the in vitro study, the total amount of RNA was isolated from DRG cultures using TRI-Reagent® and reverse-transcribed according to the manufacturer’s instructions (Im-Prom-II™ Reverse Transcription System, Promega, WI, USA). For the in vivo study, samples were taken and prepared for the experiments as described^3^. RT-qPCRs were performed in triplicate on 96-well plates (CFX96™ Real-Time PCR Detection Systems, Bio-Rad Inc., Milano, Italy) and the fluorescence intensity was assessed using the CFX96™ Real-Time PCR Detection Systems (Bio-Rad Inc.). The initial denaturation step was set at 95 °C for 10 min, followed by 40 cycles of amplification using the following primers: mouse Slc9A1 5′-CATCCTTGTCTTCGGGGAGT3′, forward; 5′-ACCACGAAGAAGCTCAGGAA-3′, reverse; the annealing temperature was 60 °C. The transcripts were normalized to the expression of ribosomal protein S18 mRNAs and the relative threshold cycle (∆Ct) was calculated. The ∆Ct of treated cells was compared to the ∆Ct generated by control cells (untreated), and log_2_ fold change was calculated as the difference between them (log_2_FC =  − ∆∆Ct).

### Statistical analysis

Data were tested for homoscedasticity and residual normality (Levene’s and Shapiro–Wilk tests, respectively). When dataset values were normal-distributed and showed homoscedasticity, mean (M) ± standard error of the mean (SEM) were used as measures of central tendency and dispersion, and parametric tests were used as detailed in the text. Otherwise, results were reported as mean (M), median (MD) and interquartile range (IQR) and nonparametric tests were employed. In all cases, tests were conducted at the significance level α = 0.05 (OriginPro 9.1, OriginLab, USA). Sample size for the in vivo part of the study was calculated as done in previous studies on OIPN on the basis of nerve conduction velocity reference values of our laboratory^51^, assuming that the relevant difference between CTRL and treated groups is 5 m/s (with an estimated standard deviation = 7); thus, if a 2-sided 5% alpha and a 80% power is set, the sample size is = 7 animals/group (www.dssresearch.com/KnowledgeCenter/toolkitcalculators/samplesizecalculators.aspx). Nerve conduction studies were analysed with non-parametric tests (Mann–Whitney U test). Two-sided tests were used. A p-value < 0.05 was set as significant. All analyses were conducted in GraphPad (GraphPad Inc, La Jolla, CA) environment (v4.0).

## Supplementary Information

Supplementary Table S1.

## Data Availability

The datasets generated during the current study are available from the corresponding author on reasonable request.
