# Supplementary material for: Inhibition of NHE1 transport activity and gene transcription in DRG neurons in oxaliplatin-induced painful peripheral neurotoxicity
Source: Sci Rep. 2023 Mar 9;13:3991. doi: 10.1038/s41598-023-31095-9 (PMC9998445; doi:10.1038/s41598-023-31095-9)
Supplement: Supplementary file 1 — Supplementary Table S1. [file 41598_2023_31095_MOESM1_ESM.docx]

**Supplementary Information**

**Inhibition of NHE1 transport activity and gene transcription in DRG neurons in oxaliplatin-induced painful peripheral neurotoxicity**

Marianna Dionisi^1^, Beatrice Riva^1^, Marta Delconti^1^, Cristina Meregalli^2^, Alessia Chiorazzi^2^, Annalisa Canta^2^, Paola Alberti^2^, Valentina Carozzi^2^, Eleonora Pozzi^2^, Dmtry Lim^1^, Armando A. Genazzani^1^, Carla Distasi^1^*^+^ & Guido Cavaletti^2+^

^1^Department of Pharmaceutical Sciences, Università del Piemonte Orientale, Novara, Italy.

^2^Experimental Neurology Unit, School of Medicine and Surgery, University of Milano-Bicocca, Monza, Italy.

^+^These authors contributed equally to this manuscript

*Author to whom correspondence should be addressed:

carla.distasi@uniupo.it

**Table S1:** body weight (g) of rats during the treatment period.

|  | **ANIMAL** | **DAY 1** | **DAY 4** | **DAY 7** | **DAY 11** | **DAY 14** | **DAY 17** | **DAY 22** | **DAY 25** | **DAY 30** | **DAY32** | **DAY 36** | **DAY 39** |
| --- | --- | --- | --- | --- | --- | --- | --- | --- | --- | --- | --- | --- | --- |
| **VEH** | **1** | 335,29 | 345,32 | 359,73 | 372,35 | 378,13 | 389,13 | 399,97 | 405,2 | 416,8 | 416,17 | 425,88 | 431,06 |
|  | **2** | 316,41 | 324,98 | 335,93 | 347,84 | 357,34 | 365,6 | 381,6 | 387,03 | 398,1 | 401,5 | 411,31 | 416,45 |
|  | **3** | 295,34 | 300,00 | 312,06 | 325,31 | 332,98 | 342,33 | 355,36 | 360,09 | 371,91 | 368,11 | 378,01 | 385,4 |
|  | **4** | 324,54 | 332,91 | 346,07 | 360,3 | 366,23 | 375,04 | 386,75 | 395,89 | 402,65 | 409,63 | 416,12 | 419,68 |
|  | **5** | 304,51 | 310,04 | 325,04 | 337 | 346,79 | 352,43 | 362,54 | 369,11 | 382,08 | 378,57 | 392,49 | 399,12 |
|  | **6** | 318,63 | 325,14 | 340,86 | 353,74 | 361,4 | 370,99 | 387,44 | 392,9 | 406 | 407,9 | 413,6 | 422,28 |
|  | **7** | 299,56 | 308,32 | 324,99 | 335,17 | 340,24 | 346,79 | 360,74 | 367,71 | 380,45 | 377,11 | 385,11 | 388,73 |
|  | **Mean** | **313,47** | **320,96** | **334,95** | **347,39** | **354,73** | **363,19** | **376,34** | **382,56** | **394,00** | **394,14** | **403,22** | **408,96** |
|  | **SD** | **14,35** | **15,75** | **15,78** | **16,20** | **15,69** | **16,83** | **16,79** | **16,95** | **16,17** | **19,06** | **17,94** | **17,79** |
|  |  |  |  |  |  |  |  |  |  |  |  |  |  |
| **OHP** | **8** | 297,70 | 303,35 | 308,08 | 312,63 | 307,61 | 305,21 | 303,49 | 306,74 | 301,07 | 298,38 | 302,64 | 299,66 |
|  | **9** | 316,74 | 319,66 | 329,31 | 328,87 | 327,34 | 328,00 | 331,30 | 332,68 | 315,75 | 330,66 | 332,85 | 333,63 |
|  | **10** | 350,45 | 356,40 | 368,53 | 369,25 | 363,68 | 374,57 | 378,55 | 382,79 | 384,58 | 376,08 | 390,33 | 383,25 |
|  | **11** | 305,49 | 307,27 | 314,99 | 318,66 | 313 | 310,61 | 305,35 | 306,04 | 293,29 | 258,04 | **dead** |  |
|  | **12** | 313,14 | 315,96 | 323,89 | 325,9 | 320,39 | 315,81 | 316,38 | 316,86 | 321,87 | 318,44 | 322,25 | 324,73 |
|  | **13** | 313,66 | 314,99 | 323,28 | 325,31 | 324,43 | 319,93 | 334,64 | 335,53 | 337,92 | 333,97 | 340,36 | 339,72 |
|  | **14** | 317,66 | 322,23 | 322,74 | 325,75 | 325,73 | 320,84 | 326,96 | 327,24 | 324,49 | 322,56 | 325,85 | 326,82 |
|  | **Mean** | **316,41** | **319,98** | **327,26** | **329,48** | **326,03** | **325,00** | **328,10** | **329,70** | **325,57** | **319,73** | **335,71** | **334,64** |
|  | **SD** | **16,57** | **17,37** | **19,47** | **18,38** | **18,09** | **23,07** | **25,36** | **26,20** | **29,97** | **35,98** | **29,61** | **27,47** |
|  |  |  |  |  |  |  |  |  |  |  |  |  |  |
| **5-FU** | **15** | 309,27 | 311,24 | 315,86 | 328,88 | 331,25 | 342,43 | 343,5 | 351,41 |  |  |  |  |
|  | **16** | 333,86 | 336,61 | 338,7 | 357,13 | 347,53 | 363,31 | 363,4 | 373,98 |  |  |  |  |
|  | **17** | 312,79 | 316,25 | 320,78 | 332,82 | 327,49 | 336,37 | 335,17 | 346,27 |  |  |  |  |
|  | **18** | 318,41 | 321,65 | 330,05 | 343,37 | 336,97 | 345,76 | 343,4 | 355 |  |  |  |  |
|  | **19** | 324,51 | 330,91 | 341,13 | 361,15 | 346,44 | 358,45 | 369,06 | 382,98 |  |  |  |  |
|  | **20** | 299,86 | 302,78 | 308,87 | 319,92 | 315,36 | 324,52 | 327,17 | 333,76 |  |  |  |  |
|  | **21** | 339,03 | 345,64 | 357,78 | 374,43 | 376,95 | 389,27 | 407,95 | 418,24 |  |  |  |  |
|  | **Mean** | **319,68** | **323,58** | **330,45** | **345,39** | **340,28** | **351,44** | **355,66** | **365,95** |  |  |  |  |
|  | **SD** | **13,84** | **15,01** | **16,84** | **19,63** | **19,63** | **21,16** | **27,40** | **28,41** |  |  |  |  |

**Note:** VEH, vehicle; OHP, oxaliplatin; 5-FU: 5-fluorouracil
